# Efficacy of Modified Amnion-Assisted Conjunctival Epithelial Redirection (ACER) for Partial Limbal Stem Cell Deficiency

**DOI:** 10.3390/medicina57040369

**Published:** 2021-04-10

**Authors:** Sang Beom Han, Farah Nur Ilyana Mohd Ibrahim, Yu-Chi Liu, Jodhbir S. Mehta

**Affiliations:** 1 Department of Ophthalmology, Kangwon National University School of Medicine, Kangwon National University Hospital, Chuncheon 24289, Korea; msbhan@nate.com; 2 Singapore National Eye Centre, Singapore 168751, Singapore; farahilyana237@gmail.com (F.N.I.M.I.); liuchiy@gmail.com (Y.-C.L.); 3 Singapore Eye Research Institute, Singapore 168751, Singapore; 4 Department of Ophthalmology, Yong Loo Lin School of Medicine, National University of Singapore, Singapore 119228, Singapore

**Keywords:** amniotic membrane, amniotic membrane transplantation, limbal stem cell, limbal stem cell deficiency, limbal stem cell transplantation

## Abstract

*Background and objectives*: the aim of this study was to analyze the efficacy of a modified “amnion-assisted conjunctival epithelial redirection (ACER)” technique for the treatment of partial limbal stem cell deficiency (LSCD). *Materials and methods*: the medical records of three patients with partial LSCD who underwent corneal surface reconstruction with modified ACER following superficial keratectomy were retrospectively studied. Briefly, in this technique, an inner amniotic membrane (AM) layer was applied on the corneal surface to promote corneal re-epithelialization. The outer AM layer was applied as a barrier to prevent the invasion of conjunctival epithelial cells into the cornea before the corneal surface was completely covered by corneal epithelial cells derived from the remaining intact limbal stem cells. *Results:* in all three cases, the outer AM layer successfully kept the conjunctival epithelium away from the corneal surface and prevented an admixture of conjunctival epithelial cells with corneal epithelial cells. In all three patients, the cornea was completely re-epithelized with epithelial cells derived from the remaining healthy limbal stem cells, and a clear visual axis was maintained without recurrence for a mean follow-up period of 37.3 ± 8.6 months. *Conclusions:* the preliminary results suggest that modified ACER appears to be a viable option for patients with partial LSCD.

## 1. Introduction

Limbal stem cells (LSCs) play an important role in the replenishment of corneal epithelial cells, which is important for the maintenance of corneal transparency [1,2,3].

Destruction or dysfunction of LSCs can lead to limbal stem cell deficiency (LSCD) [1,2], which is characterized by conjunctivalization of the cornea, corneal neovascularization, persistent corneal epithelial defect, and corneal stromal scarring and opacity [1,3,4]. Hence, it is one of the main causes of corneal blindness associated with ocular surface failure [1,3,4,5,6]. LCSD can be caused by various etiologies, such as, trauma, infection, chemical/thermal burn, congenital abnormalities, contact lens wear, and cicatricial inflammation including mucous membrane pemphigoid and Stevens–Johnson syndrome [3].

The treatment of LSCD is challenging and often requires transplantation of limbal tissue or cultivated LSCs [3,6,7,8]. Limbal stem cell transplantation (LSCT) has been widely used for the recovery of limbal function and the promotion of corneal surface reconstruction with limbal-derived epithelial cells, particularly for total LSCD [8,9]. Autologous LSCT techniques including simple limbal epithelial transplantation (SLET) and conjunctival-limbal autograft (CLAU) have shown high success rates with low risk of complications [8,9,10]. SLET may also be a less expensive and easier technique compared to other treatment options [10]. However, it is often difficult to harvest a sufficient amount of autologous limbal tissue, especially in bilateral cases [1,11]. Although allogenic LSCT techniques, such as, keratolimbal allograft (KLAL) or living-related limbal conjunctival allograft (lr-CLAL), are more advantageous for providing a large amount of limbal stem cells, these are associated with a risk of allograft rejection and need immunosuppression [1,8]. For partial LSCD, LSCT may also be performed for restoration of limbal function and re-epithelialization of the corneal surface [8,9]. However, it can also be associated with the above drawbacks [1,8,11].

Transplantation of an amniotic membrane (AM) has been effectively performed in conjunction with LSCT for both total and partial LSCD [12,13,14,15], as AM can provide mechanical and biological support to the transplanted LSCs [3,16]. AM has anti-inflammation, antibacterial, antiangiogenesis, and antifibrotic effects and can promote the migration and adhesion of limbal epithelial cells [16]. Thus, AM can function as a biologic bandage contact lens that can promote expansion of the remaining intact LSCs [1,17,18].

In less severe cases of partial LSCD, sequential sector conjunctival epitheliectomy (SSCE), alone or in combination with AMT, can be effective for the prevention of conjunctival epithelial ingrowth [3,6,7,19,20,21]. In brief, abnormal conjunctival tissue on the cornea is mechanically scraped back to the bulbar conjunctival surface repeatedly until the corneal surface is re-epithelialized with limbal-derived corneal epithelial cells [6,20,21]. However, the method has not been widely adopted in that it often requires multiple treatments to prevent the recurrence of conjunctival ingrowth as well as causes pain and bleeding from surface vessels [6,7,20,21]. Several studies have suggested that AMT without LSCT may be successful for the treatment of partial LSCD over a one-year follow-up [22,23,24]. However, studies have reported that the long-term success rate of AMT does not appear to be satisfactory over a mean follow-up period of 52 months [12,25]. Moreover, AMT without limbal transplantation cannot theoretically avoid the admixture of conjunctival and corneal/limbal epithelium because migration of the conjunctival epithelial cells occurs faster than regeneration of the corneal epithelial cells without the complete recovery of limbal barrier function [7].

Recently, Dua et al. [7] introduced a procedure termed amnion-assisted conjunctival epithelial redirection (ACER), in which AM was applied to redirect the ingrowing conjunctival epithelium onto the AM. This allowed time for the corneal surface to be reconstituted from a LSCT in cases with total LSCD [7]. In the original technique, AM was used to prevent the admixture of corneal and conjunctival epithelium before corneal epithelium was completely recovered by the limbal transplantation [7].

In the present study, we aimed to introduce a procedure of modified ACER in which the outer AM layer was used to prevent the migration of conjunctival epithelium onto the cornea and the inner AM layer was used to aid corneal epithelial regeneration, and we evaluated the preliminary efficacy and safety of the procedure in patients with partial LSCD.

## 2. Patients and Methods

### 2.1. Patients

This retrospective study included three patients with partial LSCD who underwent modified ACER at Singapore National Eye Center from April to July 2017 with a follow-up of at least 24 months. Data including demographics, medical history, causes of the LSCD, best-corrected visual acuity (BCVA) before and at final visit after the surgery, and clinical findings were collected.

### 2.2. Surgical Technique for Modified ACER

**Figure 1:**
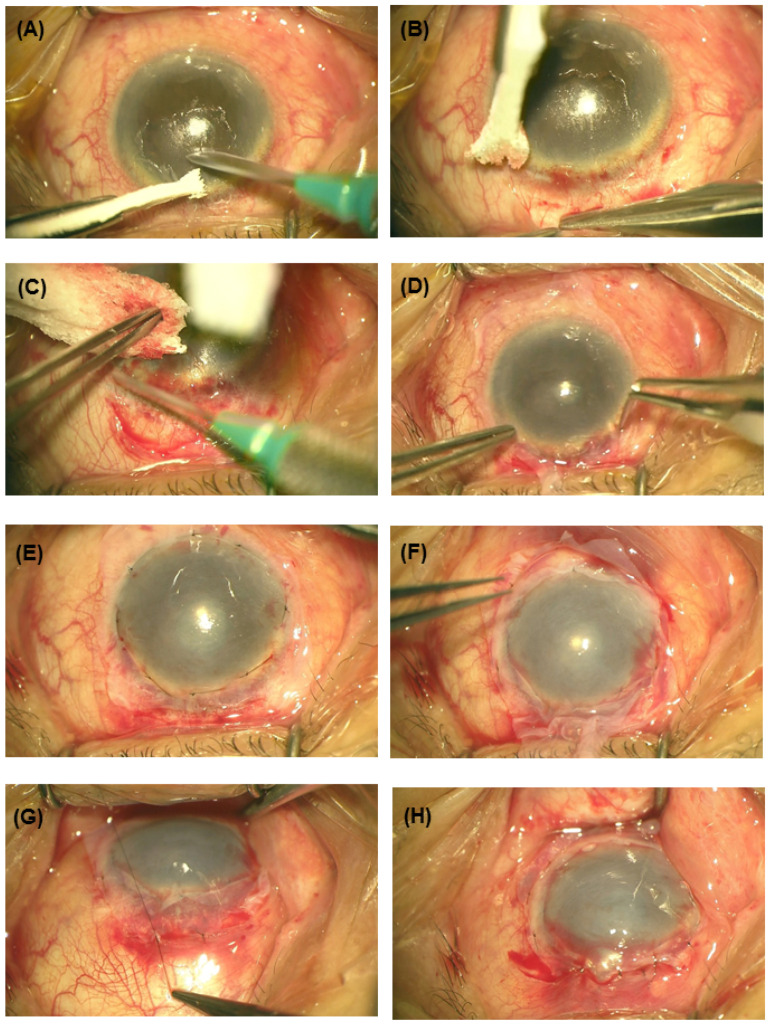
The surgical procedures of the modified amnion-assisted conjunctival epithelial redirection (ACER). (**A**) Conjunctival tissue on the cornea was dissected and peeled off using the Beaver blade. (**B**,**C**) The conjunctiva in the localized area of limbal stem cell deficiency (LSCD) was peritomized and recessed. (**D**,**E**) The inner amniotic membrane (AM) was placed and fixed onto the cornea with interrupted 10-0 nylon sutures. (**F**) The outer AM was placed on top of the inlay and the margin of the outer AM was anchored to the adjacent conjunctiva with interrupted 10-0 nylon sutures (**G**,**H**). The edge of the AM in the area of the partial LSCD was sutured to the free edge of the peritomized conjunctiva with additional interrupted 10-0 nylon sutures.

All procedures were performed under local anesthesia. After insertion of a lid speculum, the fibrovascular conjunctival tissue on the cornea was dissected and peeled off using a Beaver blade (Rudolph Beaver, Inc.; Belmont, MA, USA)) and bleeding was controlled with adrenalin swabs (Figure 1A). The conjunctiva in the localized area of LSCD was peritomized and recessed for 2–3 mm (Figure 1B,C). The second layer of AM (outer layer) was placed on top of the inner AM layer with the epithelial side up and the margin of the outer AM anchored to the adjacent conjunctiva with interrupted 10-0 nylon sutures (Figure 1F). The edge of the AM in the area of the partial LSCD was sutured to free edge of peritomized conjunctiva with additional interrupted 10-0 nylon sutures (Figure 1G,H).

Postoperatively, topical levofloxacin 0.5% and dexamethasone 0.1% were applied 4 times a day for 1 month and then twice a day for another month. Interrupted 10-0 nylon sutures were removed 1 or 2 weeks after surgery. The outer AM layer was removed at the slit lamp under topical anesthesia at 2 months postoperatively in 2 cases and spontaneously detached at 2 months in 1 case.

## 3. Results

**Figure 2:**
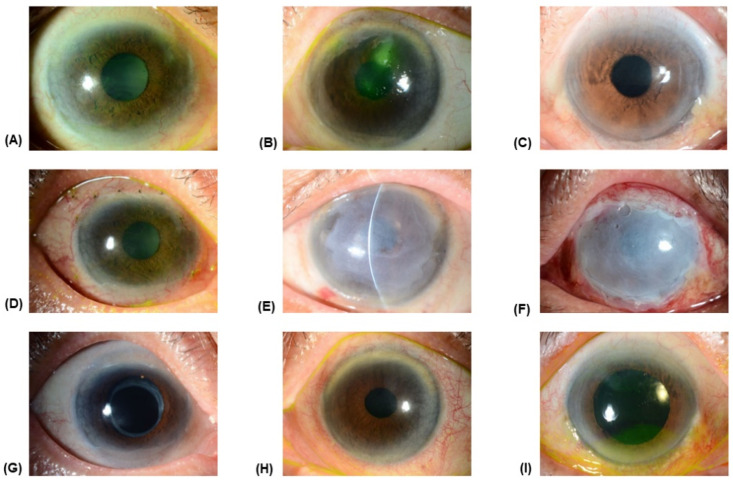
The anterior segment photographs before, at two weeks after surgery, and at the last follow-up visit. (**A**–**C**) Preoperative photographs of patients 1, 2, and 3, respectively, demonstrate partial LSCD involving visual axis. (**D**–**F**) At two weeks postoperatively, the conjunctival epithelial tissue at the site of partial LSCD migrated onto the outer AM. (**G**–**I**) All eyes maintained clear corneal epithelium at the last follow-up visit (In patient 3, a focal area of recurrence of the superficial fibrovascular pannus was observed at the superonasal area but did not affect the visual axis.).

The patient details are given in Table 1. The patients comprised two men and one woman, and the average age of the three patients was 72.0 ± 8.9 (Mean ± SD; range, 62–79) years. All three patients had partial LSCD involving corneal centers (Figure 2A–C for patients 1, 2, and 3, respectively). The causes of the partial LSCD were long-term contact lens wear, Salzmann’s degeneration, and floppy eyelid syndrome, respectively. The mean follow-up period was 37.3 ± 8.6 (Mean ± SD; range, 28–45) months.

In all three patients, the conjunctival epithelial tissue in the area of partial LSCD migrated from the peritomized conjunctiva and grew onto the outer layer of AM, which could be clearly observed at two weeks postoperatively (Figure 2D–F for patients 1, 2, and 3, respectively). After removal of the inner AM, the corneal surface was completely epithelialized. All eyes maintained clear corneal epithelium at the last follow-up visit (Figure 2G–I for patients 1, 2, and 3, respectively). In patient 3, a focal area of recurrence of the superficial fibrovascular pannus was observed at the superonasal area but did not affect the visual axis. Initial best-corrected visual acuity (BCVA) ranged from 20/70 to 20/60 and improved in all three patients, ranging from 20/40 to 20/25 (Table 1).

## 4. Discussion

In the present study, we introduced a modified ACER technique and showed the preliminary results in three patients. In all three patients, complete recovery of a stable corneal surface with improvement of visual acuity was attained, with a mean follow-up period of 37.3 ± 8.6 months.

A few studies have suggested that AMT alone could be an effective treatment option in cases with partial LSCD [22,23,24]. Sharma et al. [22]. reported that AMT alone was not inferior to AMT with cultivated LSCT in the anatomical outcome in cases with partial LSCD caused by ocular chemical injury. However, in their study, the LSCs cultivated on AM were placed on the cornea; thus, the LSCs were not used for reconstruction of the limbal tissue [22]. Anderson et al. [24] demonstrated that superficial keratectomy in combination with AMT might to be safe and effective in restoring a stable corneal epithelium in cases of partial LSCD. Kheirkhah et al. [23] also showed that superficial keratectomy followed by AMT using fibrin glue could be an effective and safe method of recovering clear and stable corneal epithelium for cases with partial LSCD, with 120 to almost 360 degrees during a mean follow-up period of 14.2 ± 7.7 months (range 6 to 26 months) [23]. However, although AMT appears to be effective in achieving corneal re-epithelialization, it might have limited long-term efficacy for maintaining a clear and stable corneal epithelium [3,25]. Konomi et al. [25] showed that the success rate of AMT combined with superficial keratectomy in 16 cases with partial LSCD is only 40–54% at an average follow-up period of 52 months (range 12–123 months) [25]. Westekemper et al. [26] also demonstrated that AMT could not prevent partial or total LSCD, although it appeared to be effective for supporting epithelial healing after the acute phase of an ocular chemical injury [26]. AMT without LSCT for partial LSCD does not show satisfactory long-term results due to more rapid conjunctival ingrowth on the corneal surface compared to corneal re-epithelialization in cases with incomplete limbal barrier function [3,25].

In the original ACER technique recently introduced by Dua et al. [7], the outer AM layer was used as a barrier to prevent invasion of conjunctival epithelial cells migrating from the recessed conjunctiva while enabling the inner AM layer to facilitate reconstruction of the corneal surface with corneal epithelial cells derived from transplanted LSCs [7]. In LSCT, it is often difficult to harvest a sufficient amount of donor limbus tissue to cover the whole limbal area, which inevitably causes only a limited area of the limbus to be covered by transplanted limbal tissue and a wide area of the limbal region to be left uncovered and vulnerable to migration of the conjunctival epithelium onto the corneal surface [7]. As the conjunctival epithelium from the peritomized conjunctiva often grows and migrates faster compared to corneal re-epithelialization with limbal explant-derived corneal epithelial cells, prevention of the migration of the conjunctival epithelial tissue is important for successful recovery of the corneal surface [7].

We hence developed the modified ACER technique introduced in the present study based on the postulation that this technique might be beneficial for partial LSCD as the outer AM layer could function as a barrier preventing the invasion of the migration of conjunctival epithelial cells from the recessed conjunctiva while the inner AM layer can promote re-epithelialization of the corneal surface derived from the remaining healthy limbal tissue after superficial keratectomy. Our modified ACER appears to be an effective surgical method to overcome this issue and the challenges associated with SSCE, hence improving the long-term prognosis of AMT following superficial keratectomy without LSCT. As the modified technique does not involve the transplantation of limbal tissue, it is not associated with the risk of immune rejection. Our preliminary results show that the modified ACER combined with superficial keratectomy might be safe and effective for the treatment of partial LSCD, with good long-term outcomes.

The limitations of this study are as follows: (1) The modified ACER may be effective only in cases with a sufficient amount of remaining intact LSCs, as corneal epithelial defect after superficial keratectomy must be covered by corneal epithelial cells derived from the remaining healthy LSCs. LSCT might be necessary for the restoration of a clear and stable corneal surface in cases without enough support from the remaining LSCs (2) This study included preliminary data of only three patients, although there is good long-term follow up to assess the efficacy. We believe further studies with larger patient group are needed. (3) There is a risk of spontaneous detachment of the outer AM layer or dehiscence of the sutures that can lead to migration of conjunctival epithelial cells onto the corneal surface. However, this can be prevented by adjunctive measures, such as the application of fibrin glue for firm attachment of the outer AM layer [27]. (4) As AM is not a completely transparent tissue, it might often be difficult to monitor the corneal re-epithelialization covered by the outer AM layer [7]. However, technological developments, such as in vivo confocal microscopy, will enable the visualization of corneal epithelial cells under the outer AM layer [28].

In conclusion, we introduced a modified ACER technique combined with superficial keratectomy for the treatment of partial LSCD. The preliminary results showed good long-term outcome, suggesting that the modified ACER might be a useful option for the management of partial LSCD patients, obviating the need of LSCT and decreasing the risk associated with immunosuppression.

## Figures and Tables

**Table 1:** Clinical data of three patients who received modified Amnion-Assisted Conjunctival Epithelial Redirection (ACER).

Patient	Age	Sex	Race	Cause of Partial LSCD	Preop BCVA	Final PostOp BCVA	Ocular Comorbidity	Follow-Up (mo)
1	62	M	Chinese	Contact lens wear	20/60	20/25	Angle closure glaucoma (previous laser peripheral iridectomy)	28
2	79	F	Chinese	Salzmann’s degeneration	20/60	20/40	None	45
3	75	M	Chinese	Floppy eyelid syndrome	20/70	20/30	Pseudophakia	39

## Data Availability

The data presented in this study are available on request from the corresponding author. The data are not publicly available due to privacy of the study subjects.
